# Importance of IL-10 Modulation by Probiotic Microorganisms in Gastrointestinal Inflammatory Diseases

**DOI:** 10.5402/2011/892971

**Published:** 2011-02-08

**Authors:** Alejandra de Moreno de LeBlanc, Silvina del Carmen, Meritxell Zurita-Turk, Clarissa Santos Rocha, Maarten van de Guchte, Vasco Azevedo, Anderson Miyoshi, Jean Guy LeBlanc

**Affiliations:** ^1^Centro de Referencia para Lactobacilos (CERELA-CONICET), Chacabuco 145, San Miguel de Tucumán, T4000ILC Tucumán, Argentina; ^2^Institute of Biological Sciences, Federal University of Minas Gerais (UFMG-ICB), Belo Horizonte, MG, CEP 31270-901, Brazil; ^3^Microbial Genetics Research Unit, Microbiology and the Food Chain Department, INRA Research Centre of Jouy-en-Josas, 78352 Paris, France

## Abstract

Lactic acid bacteria (LAB) represent a heterogeneous group of microorganisms that are naturally present in many foods and possess a wide range of therapeutic properties. The aim of this paper is to present an overview of the current expanding knowledge of one of the mechanisms by which LAB and other probiotic microorganisms participate in the prevention and treatment of gastrointestinal inflammatory disease through their immune-modulating properties. A special emphasis will be placed on the critical role of the anti-inflammatory cytokine IL-10, and a brief overview of the uses of genetically engineered LAB that produce this important immune response mediator will also be discussed. Thus, this paper will demonstrate the critical role that IL-10 plays in gastrointestinal inflammatory diseases and how probiotics could be used in their treatment.

## 1. Lactic Acid Bacteria

Lactic acid bacteria (LAB) constitute a phylogenetically heterogeneous group of ubiquitous microorganisms that are naturally present in media rich organic products, such as foods and occupy a wide range of ecological niches ranging from the surface of plants to the gastrourogenital tract of animals. Currently, the LAB group includes a large number of cocci and bacilli, such as species of the genera *Carnobacterium, Enterococcus, Lactobacillus, Lactococcus, Leuconostoc, Oenococcus, Pediococcus, Streptococcus, Tetragenococcus, Vagococcus* and *Weissella*, all presenting a G+C content in their DNA lower than 55%. Although quite diverse, the members of this group have various characteristics in common, that include being (i) gram-positive, (ii) facultative anaerobes, (iii) nonsporulating, (iv) nonmotile, and (v) possess the capacity to convert sugars into lactic acid [1, 2]. The first genome of LAB (*Lactococcus lactis* ssp. *lactis* IL1403) was sequenced and published in 2001. To date, 31 complete LAB genomes have already been sequenced, annotated, and published, while 67 projects are currently ongoing [3].

LAB are one of the most important industrial groups of bacteria, being widely used in food production, health improvement, and production of macromolecules, enzymes, and metabolites [4]. Since the 1980's, many efforts have been made to better understand the molecular basis of LAB's technological properties in order to control the industrial processes involving these important microorganisms.

LAB have been used since ancient times in food fermentation processes and preservation. Due to their lack of pathogenicity, most LAB species, excluding some pathogenic microorganisms such as *Streptococcus pneumonia*, are considered to be “GRAS” (generally recognized as safe) by the US Food and Drug Administration. In addition to their important technological properties in food production (production of lactic acid, decrease of lactose, improvement of organoleptic, and physical characteristics), various species of LAB, such as *Lactobacillus casei*, *Lactobacillus delbrueckii*,* Lactobacillus acidophilus*, *Lactobacillus plantarum*, *Lactobacillus fermentum,* and *Lactobacillus reuteri*, have been shown to possess therapeutic properties since they are able to prevent the development of some diseases as shown mostly using animal models [5], they have the capacity to promote beneficial effects in human and animal health and [6], are therefore, referred to as being “probiotic.” This term was popularized by R. Fuller in 1989 and was defined as “living microorganisms which upon ingestion in adequate quantities confer a benefit to the host's health” [7]. Probiotic organisms can exert their effects locally or during transient passage through the gastrointestinal system [6], and the most common means of administration is still their inclusion in fermented dairy products.

Some of the health benefits which have been claimed for probiotics include improvement of the normal microbiota, prevention of infectious diseases and food allergies, reduction of serum cholesterol, anticarcinogenic activity, stabilization of the gut mucosal barrier, immune adjuvant properties, alleviation of intestinal bowel disease symptoms, and improvement of the digestion of lactose in intolerant hosts [8]. The most commonly used strains as probiotics are members of lactobacilli, enterococci, and bifidobacteria groups [6].

Although some of these therapeutic applications have been questioned, growing concerns about health and well-being, along with an interest in consuming natural foods, have drawn considerable attention to probiotics, especially in the dairy products industry. Currently, many milk products containing probiotics are available on the market [9]. The specific health effects of selected probiotic strains are becoming increasingly accepted and relief of lactose intolerance symptoms, shortening of rotavirus diarrhea, and treatment of allergies are now well established. However, many proposed beneficial effects of probiotics still need further research, and more information about their mechanisms of action are needed before they can be considered for the prevention and treatment of specific diseases [6].

Besides their use as probiotics, LAB have potential use for new applications, such as the production of heterologous molecules of medical and biotechnological interest, such as cytokines, enzymes, and antigens in bioreactors, in fermented food products or directly in the digestive tract of humans and other animals [1]. The most promising new application for LAB is their use as live delivery vectors for antigenic or therapeutic protein delivery to mucosal surfaces. Such engineered LAB are able to elicit both mucosal and systemic immune responses. Efficient expression systems have already been developed for controlled and targeted production of desired antigens to be presented to the gastrointestinal mucosal immune system [10–12]. Currently, the majority of vehicles used for vaccine delivery are derived from pathogenic microorganisms; nevertheless, and despite using attenuated strains, the risk associated with possible reversion of these pathogens to a virulent phenotype is a major concern [13]. The use of LAB as DNA delivery vehicles represents an attractive alternative to the use of such attenuated pathogens.

## 2. Interleukin-10

Interleukin-10 (IL-10) is a pluripotent cytokine and the most important anti-inflammatory cytokine found within the human immune response [14]. IL-10 was first described as a product of T-helper type 2 (Th2) cells that inhibited cytokine synthesis in Th1 cells, and receiving as such the designation of cytokine synthesis inhibition factor (CSIF) [15–17]. IL-10 is now known to be produced by numerous immune cell populations such as macrophages, monocytes, dendritic cells (DC), B cells, as well as Th2, Th1, CD4+CD25+ naturally occurring regulatory T cells (nTreg), Tr1 and CD8^+^ cells [18, 19] and as such, modulates the function of several adaptive immunity-related cells. IL-10 can induce development of IL-10-producing T cells by acting on antigen-presenting cells (APCs) such as dendritic cells and/or directly on the Treg cells. Tolerogenic DC can influence the development and activity of regulatory T cells [18]. Furthermore, receptors for IL-10, IL-10R1, and IL-10R2, are expressed on many cell types and can also be observed in IL-10-producing cells, suggesting that the IL-10-secreting cells can themselves also be targets [18]. However, IL-10 is also produced by nonimmune cell sources such as keratinocytes, epithelial cells, and even tumor cells [18, 20].

Human IL-10 is a homodimer with a molecular mass of 37 kDa and each monomer consists of 160 amino acids. The human IL-10 gene is located on chromosome 1 and encodes for five exons [21]. Human and murine IL-10 sequences exhibit a 80% homology approximately [14].

IL-10 exerts a profound effect on immune responses having the ability to differently affect the function of several immune cell subsets and is, therefore, considered to be a broad effector molecule in immunoregulation/host defense [14]. IL-10 is generally considered an immunosuppressive molecule with its main biological function being the limiting and termination of inflammatory responses and the regulation of differentiation and proliferation of several immune cells such as T cells, B cells, natural killer cells, APCs, mast cells, and granulocytes [14]. However, IL-10 also mediates immunostimulatory properties in several *in vitro* and *in vivo* models [21]. The balance between both immunostimulatory and immunosuppressive effects is greatly influenced by the dominant cell function determining a given immune phenomenon. Thus, IL-10 can directly regulate innate and adaptive Th1 and Th2 responses by limiting T cell activation and differentiation in the lymph nodes as well as suppressing proinflammatory responses in tissues, leading to impaired pathogen control and/or reduced immunopathology [22]. 

As such, all the activities of IL-10 affects the inflammatory or specific cellular immune response (Th1, cytokine proinflammatory secretion by macrophages, and modulation of Th2) and stimulates functions of innate immunity (NK cell activity, noninflammatory removal of particles, cells, and microbes by stimulating phagocytosis) and of Th2 related immunity. Thus, it inhibits the production of proinflammatory mediators while enhancing the production of anti-inflammatory mediators [14].

IL-10 has an effect on survival, proliferation, and differentiation of human B cells as well as inducing IgA and IgG production by B cells. Regarding the effects on T cells, IL-10 inhibits the production of IL-2 and IFN-*γ* by Th1 cells [23, 24] and decreasing T cell-mediated immunity while enhancing humoral immune response. IL-10 also promotes antigen uptake by DC [25, 26], inhibits DC migration [27, 28], and increases the expression of toll-like receptors (TLR) on monocyte lineage cells [29, 30]. Moreover, IL-10 also stimulates NK cell cytotoxicity [21, 31]. The effects of IL-10 on immune cells suggest that the major physiological importance of IL-10 seems to be the limitation of inflammation, prevention of uncontrolled unregulated immunological reactions as well as the support of the humoral (Th2) immune response [24]. The powerful immunomodulatory properties of IL-10 and the promising results from IL-10 delivery on the course of several inflammatory diseases in experimental models induced the interest on clinical application of IL-10. However, inadequate IL-10 expression seems to have a considerable pathological impact. Both overexpression (e.g., in lymphoma) as well as IL-10 deficiency (e.g., in inflammatory bowel disease) are likely to have a physiological significance. Therefore, neutralization of the cytokine could be a promising approach to treat diseases from the first group whereas application of IL-10 itself could be helpful for diseases from the second group [14]. It is believed that the reliable manipulation of immune responses by controlling IL-10 expression in the cellular location where it is produced may someday become reality [32]. In this context, the aim of this review is to present an overview of the current expanding knowledge of one of the mechanisms by which LAB and other probiotic microorganisms participate in the prevention and treatment of gastrointestinal inflammatory disease through their immune-modulating properties with special emphasis on the critical role of the anti-inflammatory cytokine IL-10.

## 3. Immunomodulatory Properties of Probiotics

The intestinal mucosa is the body's first line of defense against pathogenic and toxic invasions from food. After ingestion, orally administered antigens encounter the GALT (gut associated lymphoid tissue), which is a well-organized immune network that protects the host from pathogens and prevents ingested proteins from hyperstimulating the immune response through a mechanism called oral tolerance [33]. 

The main mechanism of protection given by the GALT is humoral immune response mediated by secretory IgA (s-IgA) which prevents the entry of potentially harmful antigens, while also interacting with mucosal pathogens without potentiating damage. The stimulation of this immune response could thus be used to prevent certain infectious diseases that enter the host through the oral route. An increasing number of probiotic strains have shown to highly increase s-IgA, therefore the stimulation of IgA-producing cells is often considered a must in probiotic screening trials [34].

The use of pathological models, such as inflammatory bowel disease (IBD) animal models have proven useful in the elucidation of the immune mechanisms involved in pathogenesis. For example, it has been shown that a deregulation of T cells, caused by an imbalance between Treg and Th1, Th2, and Th17 can cause an excessive production of effector T cells that can in turn participate in the development and exacerbation of IBD [35, 36]. Murine models of IBD have demonstrated that CD4+ T cell differentiation plays a pivotal role in determining the type of immune response generated in the gut and that distinct cytokine profiles characterize each CD4+ T cell subset (Th1, Th2, Th17, and Tr) [37–39]. In Crohn's disease (CD) patients, an abnormal amount of CD4+ lymphocytes with Th1 phenotype are present whereas in patients with ulcerative colitis (UC) there is an imbalance towards the Th2 phenotype [40]. New immunological insights implicate Th17 cells in the pathogenesis of CD and the importance of Th1 and Th17 cells as targets to treat this pathology [41]. These activated cytotoxic T cells exacerbate the inflammatory process through the release of proinflammatory cytokines and chemokines upon lysis of epithelial cells and the increased influx of luminal antigens at the site of epithelial erosions [42].

The tolerogenic effect of the gut microbiota may partially be mediated by the generation of regulatory T cells (Treg) or through the stimulation of Th2 cell populations which promote oral tolerance induction, preventing hypersensitivity and local inflammation [43, 44]. It was shown that *L. rhamnosus* GG reduced the number of activated T lymphocytes in the lamina propria of CD mucosa, impeding the release of IL-6 and TNF-*α* and lowering the expression of the antiapoptotic protein Bcl-2 [45].

Numerous studies have shown that certain strains of lactobacilli and bifidobacteria can modulate the production of cytokines (mediators produced by immune cells) that are involved in the regulation, activation, growth, and differentiation of immune cells. These probiotic microorganisms are able to prevent and treat certain inflammatory diseases in the gastrointestinal tract through the repression of proinflammatory cytokines. One of the central transcription factors mediating inflammatory responses is the nuclear factor *κ*B (NF-*κ*B). NF-*κ*B is required for the transcriptional activation of a number of inflammatory effectors, including IL-8, TNF-*α*, IL-6, Cox2, iNOS, and many others, and its deregulation has been detected in many inflammatory conditions. It was shown that a number of LAB can suppress inflammatory signals mediated by NF-*κ*B. These include strains of the phylogenetically closely related species *L. acidophilus* and *L. johnsonii*, which have been isolated from the human GI tract and form part of the acidophilus complex. Recent studies revealed that dairy *L. delbrueckii* strains that also belong to the acidophilus complex and that are used in yoghurt and cheese making also inhibit NF-*κ*B activation in a strain-dependent manner in human intestinal epithelial cells *in vitro* [C. Santos Rocha et al., in preparation].

These results are in agreement with the observation that the administration of probiotic yoghurt, containing live *L. delbrueckii* and *Streptococcus thermophilus*, can modulate the immune response, inducing downregulation of inflammatory cytokines (IL-17 and IL-12) produced by the immune cells involved in the inflammatory process [46]. The association of toll-like receptor (TLR) signaling deregulation and the pathogenesis of IBD infer the therapeutic potential of modulating TLR signaling pathways. Considering that probiotic bacteria and probiotic fermented products exert their effects mainly through stimulation of the innate immune response [8, 47, 48], changes in TLR expression can be associated to the beneficial effect of these microorganisms in hosts with IBD [49, 50]. Tolerance of the intestinal epithelium against bacterial ligands of the intestinal lumen is mediated by TLRs, which belong to a family of pattern-recognizing receptors that detect conserved molecular products of microorganisms emerging as crucial elements in the activation of innate immunity as well as connectors between the innate and acquired immunity. TLR4 recognizes the LPS present in the membrane of Gram (−) bacteria such as the *Enterobacteriaceae *family which tends to overgrow in an inflammatory process. In the colonic mucosa of IBD patients, high concentrations of *Enterobacteriaceae *and bacteroides were associated with severe inflammation and TLR4 increase [51]. Several studies have shown that inflammatory cytokines such as IFN-**γ**and TNF-**α**increase the expression of TLR4 and MD-2 (myeloid differentiation protein 2), resulting in increased LPS responsiveness [52]. In this context, it was shown that probiotic bacteria such as *L. casei *CRL 431 can induce changes in the TLR expression in immune and intestinal epithelial cells [53]. *L. casei* DN-114 001 prevented the development of acute DSS-induced colitis in TLR4 KO mice by inhibiting myeloperoxidase activity and IL-12p40, and increasing TGF-*β* and IL-10 mRNA. These effects suggest that the mechanism of action of  *L. casei *DN-114 001 depends on other factors besides TLR4 status [54]. 

Although, by definition, probiotics should be administered as live microorganisms, isolated components of probiotic cells may also have therapeutic benefits. It was reported that bacterial DNA has a potent immunostimulatory effect since it could attenuate colitis in a number of induced and spontaneous murine models [55]. TLR9 recognizes unmethylated CpG sequences in DNA molecules. The importance of this receptor in the etiology of IBD was observed in TLR9-deficient mice, where the intestinal inflammation was significantly lower and proinflammatory cytokine production was drastically reduced [56]. In this sense, bacterial DNA derived from luminal bacteria contributes significantly to the perpetuation of chronic intestinal inflammation through TLR9. Intestinal epithelial cells recognize pathogenic bacterial DNA and respond by increasing surface localization and expression of TLR9, leading to the secretion of the proinflammatory cytokine IL-8 [57]. Furthermore, the presence of TLR9 is also associated with the beneficial effect exerted by probiotics against IBD. It was reported that the inhibition of experimental colitis by probiotics was not observed in mice deficient in MyD88 or TLR9 [58].

In addition, it was also shown that the attenuation of DSS colitis could be caused by DNA of the VSL#3 probiotic mixture through TLR9 signaling, and nonviable bacteria were equally effective in reducing inflammation in this model [59]. Heating *E. coli* strain Nissle 1917 and its isolated DNA were also administered in a DSS murine colitis model and an anti-inflammatory effect was demonstrated [60]. Interestingly, specific immunostimulatory DNA sequences have also been shown to attenuate the production of proinflammatory cytokines which are elevated in the mucosa of UC patients, suggesting that the animal model data may be applicable to human disease states [61].

## 4. Interleukin-10 Modulation by Probiotics

There is no doubt that IL-10 plays a central role in downregulating inflammatory cascades by suppressing the secretion of proinflammatory cytokines. The crucial role of IL-10 in the prevention of IBD has been demonstrated by experiments in IL-10-deficient mice. These animals develop a chronic bowel disease resembling CD in humans, which is in part caused by a loss of suppression of the mucosal immune response toward the normal intestinal microbiota [62]. Unfortunately, systemic IL-10 treatment of CD patients is not very effective in inducing clinical remission and is associated with considerable side effects, which are partly due to the fact that high doses of systemic IL-10 induce the proinflammatory cytokine IFN-*γ* [63]. However, studies in experimental models suggest that topical treatment with IL-10 is effective in preventing certain inflammatory diseases. Recent studies have reported an accumulation of Foxp3+ CD4+ CD25+ cells in colon samples from patients with UC or CD and that subsets of IL-10-producing CD4+ CD25+ T cells were present mainly within the intestinal lamina propria, suggesting that chronic intestinal inflammation is not simply a consequence of the absence of Foxp3+ CD4+ Treg cells at the inflammation site [64]. One of the ways by which probiotics can exert immunomodulatory activities is by increasing IL-10 production that can in turn help in preventing certain IBD that are caused by abnormal inflammatory responses. However, not all probiotic strains act in the same manner. Anti-inflammatory effects, such as stimulation of IL-10-producing cells, are strain-dependent traits, and their effectiveness also depends on the concentrations used and the method of administration. Some examples of probiotic strains and mixtures or fermented foods that exert anti-inflammatory effects through the regulation of IL-10 expression are given in Table 1.

Most people think of probiotics as microorganisms that must be consumed in a food matrix; this is due to the fact that the oral administration of probiotics is the most common method of ingesting these beneficial microorganisms. Oral administration of a probiotic mixture that consisted of *Bifidobacterium longum* Bar 33 and *L. acidophilus* Bar 13 prevented inflammation and mucosal ulcerations in a trinitrobenzene sulfonic acid- (TNBS-) induced colitis mouse model [82]. This protection was associated with an upregulation of IL-10 that caused an inhibition of the TNBS-induced increase of the CD4+ population, downregulation of IL-12, and a different pattern of Foxp3+ CD4+ CD25+ cells in the intraepithelial and lamina propria lymphocytes [83]. In a human clinical trial, UC patients that were given the probiotic mixture BIFICO showed a lower recurrence level of UC flare-ups [65]. This orally administered probiotic mixture inhibited NF-*κ*B activation, decreased the expression of TNF-*α* and IL-1*β*, and increased the expression of IL-10. 

Continuing in this train of thought, it is logical to assume that fermented foods containing probiotic microorganisms could also be effective in inducing an immunostimulating/anti-inflammatory effect. It was shown that a yoghurt produced with a pool of potentially probiotic LAB strains was effective in inhibiting the propagation of a dimethylhydrazine- (DMH-) induced colon cancer in mice by increasing the number of IL-10-secreting cells, increasing cellular apoptosis and decreasing procarcinogenic enzymes [70]. Probiotics can also be added to nondairy-based foods. For example, fermented Maesil (*Prunus mume*) containing probiotics was able to suppress the development of atopic dermatitis-like skin lesions, and caused an increase in serum concentration of IL-10 and decrease of serum IL-4, eosinophil ratio, and IgE [71].

Other forms of probiotic administration have also been shown to be effective in stimulating IL-10 production. A recent study demonstrated that the rectal administration of *L. casei* DG could modify the intestinal microbiota composition and TLR expression, and increase IL-10 levels in the colonic mucosa of patients with mild UC [72]. Intranasal administration is also a popular form of probiotic administration, especially when their effects in lung tissue or against airborne pathogens are to be evaluated. The intranasal administration of the VSL#3 probiotic mixture was shown to significantly reduce IL-13 and IL-4 mRNA expression and increase IL-10 expression in lung tissues, modulating the development of a Th2-biased response [67]. An enhanced immune response to pneumococcal infection was observed in malnourished mice nasally treated with *L. casei* CRL 431 as demonstrated by an improved production of TNF-*α*, IL-4, IL-10 and IgA in the respiratory tract [73].

## 5. IL-10-Producing LAB

LAB are potential candidates to be used as vehicles for the production and delivery of heterologous proteins of vaccinal, medical, or technological interest and various delivery systems are now available for these probiotic microorganisms [9]. Different genetic engineering strategies in LAB have been used to improve their carbohydrate-fermenting properties (lactose, galactose), increase specific metabolite production (diaceyl, acetoin), produce or increase enzymatic activities (proteolytic enzymes, *α*- and *β*-galactosidase, *α*-amylase), or conferring them the capacity to produce beneficial compounds such as bacteriocins, exopolysaccharide (EPS), and other sugars, vitamins, antioxidant enzymes, and anti-inflammatory cytokines [84].

Genetically modified* Lactococcus *(*Lc.*)* lactis* secreting IL-10 provides a novel therapeutic approach for IBD. The first description of *Lc. lactis *that can secrete biologically active IL-10 was published over ten years ago [85]. In this pioneer study, murine IL-10 was synthesized as a fusion protein, consisting of the mature part of the eukaryotic protein fused to the secretion signal of the lactococcal Usp45 protein. Intragastric administration of this recombinant *Lc. lactis *strain prevented the onset of colitis in IL-10 KO mice and caused a 50% reduction of the inflammation in DSS-induced chronic colitis [81].

The application of IL-10 producing LAB is not only limited to the treatment of IBD. It was recently shown that treatment of asthma with a *Lc. lactis* expressing murine IL-10 was efficient since this LAB modulated experimental airway inflammation in the mouse model [80]. *Lc. lactis* producing recombinant IL-10 used in this study was efficient in suppressing lung inflammation, independently of Treg cells, since this cytokine plays a central role in the regulation of inflammatory cascades, allergen-induced airway inflammation, and nonspecific airway responsiveness [86]. In another study, it was shown that oral administration of an IL-10-secreting *Lc. lactis* strain could prevent food-induced IgE sensitization in a mouse model of food allergy [87]. These studies confirm that IL-10-secreting LAB hold potential for the treatment of many inflammatory diseases where this cytokine acts as a modulating compound.

Although a clear positive effect of these recombinant strains has been demonstrated, the exact mechanism by which the beneficial effect of the IL-10-producing *Lc. lactis* on the mucosa is produced remains unclear. A recent study has demonstrated the uptake of IL-10-secreting *Lc. lactis* by the paracellular route in inflamed mucosal tissue in mouse models of chronic colitis, suggesting that IL-10 production by these LAB residing inside the mucosa in the vicinity of responsive cells can improve the local action of IL-10 in inflamed tissue and the efficiency of the treatment [88]. In another study, it was shown that genetically engineered *Lc. lactis* secreting murine IL-10 could modulate the functions of bone marrow-derived DC in the presence of LPS [89]. This data suggest that the beneficial effects of IL-10 secreting LAB during chronic colitis might involve inhibition of CD4+ Th17 cells and a reduced accumulation of these cells as well as other immune cells at the site of inflammation.

## 6. Probiotics and Genetically Modified Microorganisms

Although there is no scientific evidence that supports the notion that genetically modified (GM) foods or microorganisms are dangerous for human consumption, it is necessary to demonstrate that these are innocuous in order to alleviate the fears held by the general public associated with the use of genetically modified organisms, if we want to use designed probiotics to extend the range of applications covered by natural probiotics. Also, the proper design of GM-LAB is essential in order to eliminate the risks of dissipation in the environment and prevent the transfer of certain genes (such as antibiotic resistance genes) to other microorganisms.

The construction of a biological containment system for a genetically modified *Lc. lactis* for intestinal delivery of human IL-10 is an important step forward for the safe use of GM-LAB for human therapeutic purposes [90]. In this study, the thymidylate synthase gene of *Lc. lactis* was replaced by the human IL-10 gene, making this strain incapable of growing when deprived of thymidine or thymine. This strain does not contain any antibiotic resistance markers and because of its thymidine auxotrophy, it cannot disseminate in the environment, making it one of the safest GM strains ever engineered. This containment system was recently evaluated in CD patients, and it was shown that no adverse effects where produced after consuming this GM-LAB and that it could only be recovered in feces when thymidine was added [91]. Although only preliminary results from this phase 1 trial were obtained, the use of genetically modified bacteria for mucosal delivery of proteins is a feasible strategy in human with chronic intestinal inflammation [91].

Intragastric administration of *Lc. lactis* genetically modified to secrete IL-10 *in situ* in the intestine was shown to be effective in healing and preventing chronic colitis in mice. However, its use in humans is hindered by the sensitivity of* Lc. lactis* to freeze-drying and its poor survival in the gastrointestinal tract, reasons for which novel means for more effective mucosal delivery of therapeutic LAB are currently being developed [92–94].

## 7. Conclusions

The results of animal and some human studies demonstrate that some probiotic strains can successfully modify the mucosal immune response to modulate the levels of specific activation molecules such as cytokines. By increasing IL-10 levels and in consequence decreasing inflammatory cytokines such as TNF-*α* and IFN-*γ*, some LAB can prevent the appearance of local inflammatory diseases and could be used as an adjunct therapy with conventional treatments. However, proper multicenter randomized human clinical trials are necessary to demonstrate the effectiveness of probiotics in the treatment of IBD and other inflammatory diseases.

Although probiotic effects are a strain-dependent trait, using modern genetic engineering techniques, it is theoretically possible to obtain strains that can exert a variety of beneficial properties. For example, the introduction of antioxidant enzyme genes in current probiotic strains that have natural anti-inflammatory properties, such as the ability to modulate the immune-dependant inflammatory processes, could generate very useful strains that could be applied in the treatment of a variety of inflammatory diseases [95, 96]. These strains could also be included in treatment protocols since it has been shown that probiotics can enhance the effectiveness of traditional IBD treatments. However, before proposing the genetic modification of anti-inflammatory strains, the innate mechanisms of the potential host strains should be demonstrated in properly designed large-scale human clinical trials. These trials are essential in future studies using the engineered strains to demonstrate the differences between the native and modified strains.

The consumption of engineered strains by humans is still highly controversial due to the public perception that genetic manipulation is not “natural.” Scientists must perform well-designed studies where the results are divulged to the general population in order to inform consumers of the obvious beneficial effects these novel techniques can confer with the minimum of risk to their health and to the environment [97]. Throughout the course of history, most novel treatments have met resistance from potential benefactors, it is thus important to show that the potential benefits are highly superior to the risks for novel treatments to be completely accepted by the population as a whole.

## Figures and Tables

**Table 1:** Examples of probiotic strains/mixtures/fermented foods that exert anti-inflammatory effects through the modulation of IL-10.

	Demonstrated effect/mechanism	Host	Ref.
*Probiotic mixtures*			

BIFICO mixture	Prevention of flare-ups of chronic ulcerative colitis/inhibition of NF-*κ*B activation, decrease the expressions of TNF-*α* and IL-1*β* and increased expression of IL-10	Patients with UC	[65]

VSL#3 mixture	Prevention of autoimmune diabetes/increased production of IL-10 from Peyer's patches and the spleen, increased IL-10 expression in the pancreas	Nonobese diabetic mice	[66]

	Antiallergic effect/cytokine production by spleen cells was modulated towards a Treg/Th0 profile, increased IL-10 and IFN-*γ* production, reduction of serum specific IgG1, reduction of IL-13 and IL-4 mRNA expression, and increased IL-10 expression at lung level	Allergen-induced mice	[67]

	Improvement of colitis/increased production of IL-10 and number of regulatory CD4+ T cells bearing surface TGF-*β*a in the form of latency-associated protein	TNBS-induced mice	[68]

*L. paracasei* DSM 13434, *L. plantarum* DSM 15312 & 15313	Decreased autoimmune encephalomyelitis/attenuation of proinflammatory Th1 and Th17 cytokines followed by IL-10 induction in mesenteric lymph nodes, and involvement of IL-10 producing CD4(+)CD25(+) Treg cells	Multiple sclerosis mice	[69]

*Fermented products*			

Probiotic yogurt	Inhibition of colon tumour growth/decrease of the inflammatory response by increasing IL-10-secreting cells, cellular apoptosis, and diminishing procarcinogenic enzymes	DMH-induce mice	[70]

	Reduction of the severity of intestinal inflammation/increased levels of IL-10 in the intestines and decrease in IL-17 and IL-12 levels in addition to beneficial changes in the intestinal microbiota	TNBS-induced mice	[46]

Fermented Maesil (*Prunus mume*) with probiotics	Suppression of the development of atopic dermatitis-like skin lesions/increased serum concentration of IL-10 and decreased IL-4, decreases in eosinophil ratio and serum IgE concentration	NC/Nga mouse model	[71]

*Probiotic strains*			

*L. casei *DG	Improvement of gut microbiota/reduction of TLR4 and IL-1*β* mRNA levels and significantly increased mucosal IL-10	Patients with UC	[72]

*L. casei *CRL 431	Increased resistance against *Streptococcus pneumonia*/improved production of TNF-*α* and activity of phagocytes in the respiratory tract, IL-4 and IL-10 were significantly increased, increase in the levels of specific respiratory IgA	Malnourished mice	[73]

*L. rhamnosus* GG	Amelioration of intestinal inflammation/decreased LPS-induced cytokine-induced neutrophil chemoattractant-1 production in liver and plasma, meliorated LPS-suppressed IL-10 level in lungs and decreased IL-1b production in liver	Gastrostomy-fed rats	[74]

*L. casei *BL23	Protection against colitis/increased IL-10/IL-12 cytokine ratio	TNBS-induced mice	[75]

*L. delbrueckii *UFV-H2b20	Protection against *L. monocytogenes*/induced higher production of IL-10 in the mucosal immune system, favored and effector responses (increased production of TNF-*α* in the serum, peritoneal cavity, and gut) while preventing their immunopathological consequences	Germ-free mice	[76]

*L. salivarius* CECT5713	Improvement of gut microbiota/significant increase of NK cells and monocytes, as well as the plasmatic levels of IgM, A, and G, and the regulatory cytokine IL-10	Healthy adults	[77]

*S. cerevisiae* UFMG 905	Prevention of bacterial translocation and improvement of intestinal barrier integrity/increase of IL-10 levels	Murine intestinal obstruction model	[78]

*L. casei *DN-114 001	Prevention of induced colitis/increase in TGF-*β* and IL-10 mRNA and protein expression	DSS-induced mice	[54]

*GM-strains*			

*L. plantarum *NCIMB8826ΔDlt	Immunomodulatory properties/reduction of secretion of proinflammatory cytokines (TNF-*α*, IL-1*β*, IL-6, and IL-8), and increase in IL-10 production	TNBS-induced mice	[79]

*L. lactis *IL-10	Prevention of pulmonary inflammation and mucus hypersecretion. Decrease in eosinophil numbers, EPO activity, anti-OVA IgE, and IgG1 levels, IL-4, IL-5, CCL2, CCL3, CCL5, and CCL11	OVA-induced mice	[80]

	Reduction in colitis and histological damages of the intestines/increased IL-10 levels in the intestines	DSS-induced mice	[81]

## References

[1] Nouaille S, Ribeiro LA, Miyoshi A (2003). Heterologous protein production and delivery systems for Lactococcus lactis. *Genetics and Molecular Research*.

[2] Azevedo V, Miyoshi A Novas utilizações biotecnológicas e terapêuticas das bactérias do ácido láctico.

[3] Bolotin A, Wincker P, Mauger S (2001). The complete genome sequence of the lactic acid bacterium lactococcus lactis ssp. lactis IL1403. *Genome Research*.

[4] Pfeiler EA, Klaenhammer TR (2007). The genomics of lactic acid bacteria. *Trends in Microbiology*.

[5] LeBlanc JG, de Moreno de LeBlanc A, Perdigón G (2008). Anti-inflammatory properties of lactic acid bacteria: current knowledge, applications and prospects. *Anti-Infective Agents in Medicinal Chemistry*.

[6] Ouwehand AC, Salminen S, Isolauri E (2002). Probiotics: an overview of beneficial effects. *Antonie van Leeuwenhoek*.

[7] Report of a Joint FAO/WHO Expert Consultation on Evaluation of Health and Nutritional Properties of Probiotics in Food Including Powder Milk with Live Lactic Acid Bacteria.

[8] Maldonado Galdeano C, de Moreno de LeBlanc A, Vinderola G, Bibas Bonet ME, Perdigón G (2007). Proposed model: mechanisms of immunomodulation induced by probiotic bacteria. *Clinical and Vaccine Immunology*.

[9] Miyoshi A, Bermudez-Humaran L, Pacheco de Azevedo M, Langella P, Azevedo V, Mozzi F, Raya R, Vignolo G (2010). Lactic acid bacteria as live vectors: heterologous protein production and delivery systems. *Biotechnology of Lactic Acid Bacteria Novel Applications*.

[10] Wells JM, Wilson PW, Norton PM, Gasson MJ, Le Page RWF (1993). Lactococcus lactis: high-level expression of tetanus toxin fragment C and protection against lethal challenge. *Molecular Microbiology*.

[11] Norton PM, Le Page RWF, Wells JM (1995). Progress in the development of Lactococcus lactis as a recombinant mucosal vaccine delivery system. *Folia Microbiologica*.

[12] Le Loir Y, Nouaille S, Commissaire J, Brétigny L, Gruss A, Langella P (2001). Signal peptide and propeptide optimization for heterologous protein secretion in Lactococcus lactis. *Applied and Environmental Microbiology*.

[13] Innocentin S, Guimarães V, Miyoshi A (2009). Lactococcus lactis expressing either Staphylococcus aureus fibronectin-binding protein A or Listeria monocytogenes internalin A can efficiently internalize and deliver DNA in human epithelial cells. *Applied and Environmental Microbiology*.

[14] Asadullah K, Sterry W, Volk HD (2003). Interleukin-10 therapy—review of a new approach. *Pharmacological Reviews*.

[15] Lalani I, Bhol K, Ahmed AR (1997). Interleukin-10: biology, role in inflammation and autoimmunity. *Annals of Allergy, Asthma and Immunology*.

[16] Howard M, O’Garra A (1992). Biological properties of interleukin 10. *Immunology Today*.

[17] Opal SM, Wherry JC, Grint P (1998). Interleukin-10: potential benefits and possible risks in clinical infectious diseases. *Clinical Infectious Diseases*.

[18] Moore KW, de Waal Malefyt R, Coffman RL, O’Garra A (2001). Interleukin-10 and the interleukin-10 receptor. *Annual Review of Immunology*.

[19] Kamanaka M, Kim ST, Wan YY (2006). Expression of interleukin-10 in intestinal lymphocytes detected by an interleukin-10 reporter knockin *tiger* mouse. *Immunity*.

[20] Williams LM, Ricchetti G, Sarma U, Smallie T, Foxwell BMJ (2004). Interleukin-10 suppression of myeloid cell activation—a continuing puzzle. *Immunology*.

[21] Mocellin S, Marincola F, Rossi CR, Nitti D, Lise M (2004). The multifaceted relationship between IL-10 and adaptive immunity: putting together the pieces of a puzzle. *Cytokine and Growth Factor Reviews*.

[22] Couper KN, Blount DG, Riley EM (2008). IL-10: the master regulator of immunity to infection. *Journal of Immunology*.

[23] Del Prete G, de Carli M, Almerigogna F, Giudizi MG, Biagiotti R, Romagnani S (1993). Human IL-10 is produced by both type 1 helper (Th1) and type 2 helper (Th2) T cell clones and inhibits their antigen-specific proliferation and cytokine production. *Journal of Immunology*.

[24] de Waal Malefyt R, Abrams J, Bennett B, Figdor CG, de Vries JE (1991). Interleukin 10(IL-10) inhibits cytokine synthesis by human monocytes: an autoregulatory role of IL-10 produced by monocytes. *Journal of Experimental Medicine*.

[25] Allavena P, Piemonti L, Longoni D (1998). IL-10 prevents the differentiation of monocytes to dendritic cells but promotes their maturation to macrophages. *European Journal of Immunology*.

[26] Morel AS, Quaratino S, Douek DC, Londei M (1997). Split activity of interleukin-10 on antigen capture and antigen presentation by human dendritic cells: definition of a maturative step. *European Journal of Immunology*.

[27] Demangel C, Bertolino P, Britton WJ (2002). Autocrine IL-10 impairs dendritic cell (DC)-derived immune responses to mycobacterial infection by suppressing DC trafficking to draining lymph nodes and local IL-12 production. *European Journal of Immunology*.

[28] Wang J, Guan E, Roderiquez G, Norcross MA (1999). Inhibition of CCR5 expression by IL-12 through induction of *β*- chemokines in human T lymphocytes. *Journal of Immunology*.

[29] Flo TH, Halaas O, Torp S (2001). Differential expression of Toll-like receptor 2 in human cells. *Journal of Leukocyte Biology*.

[30] Vabulas RM, Braedel S, Hilf N (2002). The endoplasmic reticulum-resident heat shock protein Gp96 activates dendritic cells via the toll-like receptor 2/4 pathway. *Journal of Biological Chemistry*.

[31] Shibata Y, Foster LA, Kurimoto M (1998). Immunoregulatory roles of IL-10 in innate immunity: IL-10 inhibits macrophage production of IFN-*γ*-inducing factors but enhances NK cell production of IFN-*γ*. *Journal of Immunology*.

[32] Mosser DM, Zhang X (2008). Interleukin-10: new perspectives on an old cytokine. *Immunological Reviews*.

[33] Weiner HL, Gonnella PA, Slavin A, Maron R (1997). Oral tolerance: cytokine milieu in the gut and modulation of tolerance by cytokines. *Research in Immunology*.

[34] O’Sullivan DJ (2001). Screening of intestinal microflora for effective probiotic bacteria. *Journal of Agricultural and Food Chemistry*.

[35] Ruddy MJ, Wong GC, Liu XK (2004). Functional cooperation between interleukin-17 and tumor necrosis factor-*α* is mediated by CCAAT/enhancer-binding protein family members. *Journal of Biological Chemistry*.

[36] Leon F, Smythies LE, Smith PD, Kelsall BL (2006). Involvement of dendritic cells in the pathogenesis of inflammatory bowel disease. *Advances in Experimental Medicine and Biology*.

[37] Neurath MF, Fuss I, Kelsall BL, Presky DH, Waegell W, Strober W (1996). Experimental granulomatous colitis in mice is abrogated by induction of TGF-*β*-mediated oral tolerance. *Journal of Experimental Medicine*.

[38] Jonuleit H, Schmitt E, Stassen M, Tuettenberg A, Knop J, Enk AH (2001). Identification and functional characterization of human CD4(+)CD25(+) T cells with regulatory properties isolated from peripheral blood. *Journal of Experimental Medicine*.

[39] Cong Y, Weaver CT, Lazenby A, Elson CO (2002). Bacterial-reactive T regulatory cells inhibit pathogenic immune responses to the enteric flora. *Journal of Immunology*.

[40] Podolsky DK (2002). Inflammatory bowel disease. *New England Journal of Medicine*.

[41] Brand S (2009). Crohn’s disease: Th1, Th17 or both? The change of a paradigm: new immunological and genetic insights implicate Th17 cells in the pathogenesis of Crohn’s disease. *Gut*.

[42] Kappeler A, Mueller C (2000). The role of activated cytotoxic T cells in inflammatory bowel disease. *Histology and Histopathology*.

[43] Perdigon G, Medina M, Vintini E, Valdez JC (2000). Intestinal pathway of internalisation of lactic acid bacteria and gut mucosal immunostimulation. *International Journal of Immunopathology and Pharmacology*.

[44] Pessi T, Sütas Y, Hurme M, Isolauri E (2000). Interleukin-10 generation in atopic children following oral lactobacillus rhamnosus GG. *Clinical and Experimental Allergy*.

[45] Gupta P, Andrew H, Kirschner BS, Guandalini S (2000). Is Lactobacillus GG helpful in children with Crohn’s disease? Results of a preliminary, open-label study. *Journal of Pediatric Gastroenterology and Nutrition*.

[46] de Moreno de LeBlanc A, Chaves S, Perdigón G (2009). Effect of yoghurt on the cytokine profile using a murine model of intestinal inflammation. *European Journal of Inflammation*.

[47] Galdeano CM, de Moreno de LeBlanc A, Carmuega E, Weill R, Perdigón G (2009). Mechanisms involved in the immunostimulation by probiotic fermented milk. *Journal of Dairy Research*.

[48] Vizoso Pinto MG, Rodriguez Gómez M, Seifert S, Watzl B, Holzapfel WH, Franz CMAP (2009). Lactobacilli stimulate the innate immune response and modulate the TLR expression of HT29 intestinal epithelial cells in vitro. *International Journal of Food Microbiology*.

[49] Ishihara S, Rumi MAK, Ortega-Cava CF (2006). Therapeutic targeting of toll-like receptors in gastrointestinal inflammation. *Current Pharmaceutical Design*.

[50] Pimentel-Nunes P, Soares JB, Roncon-Albuquerque R, Dinis-Ribeiro M, Leite-Moreira AF (2010). Toll-like receptors as therapeutic targets in gastrointestinal diseases. *Expert Opinion on Therapeutic Targets*.

[51] Schultz M, Lindström AL (2008). Rationale for probiotic treatment strategies in inflammatory bowel disease. *Expert Review of Gastroenterology and Hepatology*.

[52] Fukata M, Abreu MT (2007). TLR4 signalling in the intestine in health and disease. *Biochemical Society Transactions*.

[53] Dogi CA, Galdeano CM, Perdigón G (2008). Gut immune stimulation by non pathogenic Gram(+) and Gram(-) bacteria. Comparison with a probiotic strain. *Cytokine*.

[54] Chung YW, Choi JH, Oh TY, Eun CS, Han DS (2008). Lactobacillus casei prevents the development of dextran sulphate sodium-induced colitis in Toll-like receptor 4 mutant mice. *Clinical and Experimental Immunology*.

[55] Rachmilewitz D, Karmeli F, Takabayashi K (2002). Immunostimulatory DNA ameliorates experimental and spontaneous murine colitis. *Gastroenterology*.

[56] Obermeier F, Dunger N, Strauch UG (2005). CpG motifs of bacterial DNA essentially contribute to the perpetuation of chronic intestinal inflammation. *Gastroenterology*.

[57] Akhtar M, Watson JL, Nazli A, McKay DM (2003). Bacterial DNA evokes epithelial IL-8 production by a MAPK-dependent, NF-kappaB-independent pathway. *The FASEB Journal*.

[58] Lee J, Rachmilewitz D, Raz E (2006). Homeostatic effects of TLR9 signaling in experimental colitis. *Annals of the New York Academy of Sciences*.

[59] Rachmilewitz D, Katakura K, Karmeli F (2004). Toll-like receptor 9 signaling mediates the anti-inflammatory effects of probiotics in murine experimental colitis. *Gastroenterology*.

[60] Rachmilewitz D, Karmeli F, Shteingart S, Lee J, Takabayashi K, Raz E (2006). Immunostimulatory oligonucleotides inhibit colonic proinflammatory cytokine production in ulcerative colitis. *Inflammatory Bowel Diseases*.

[61] Kamada N, Inoue N, Hisamatsu T (2005). Nonpathogenic Escherichia coli strain Nissle 1917 prevents murine acute and chronic colitis. *Inflammatory Bowel Diseases*.

[62] Kuhn R, Lohler J, Rennick D, Rajewsky K, Muller W (1993). Interleukin-10-deficient mice develop chronic enterocolitis. *Cell*.

[63] Tilg H, Van Montfrans C, Van den Ende A (2002). Treatment of Crohn’s disease with recombinant human interleukin 10 induces the proinflammatory cytokine interferon *γ*. *Gut*.

[64] Uhlig HH, Coombes J, Mottet C (2006). Characterization of Foxp3+ CD4+ CD25+ and IL-10-secreting CD4+ CD25+ T cells during cure of colitis. *Journal of Immunology*.

[65] Cui HH, Chen CL, Wang JID (2004). Effects of probiotic on intestinal mucosa of patients with ulcerative colitis. *World Journal of Gastroenterology*.

[66] Calcinaro F, Dionisi S, Marinaro M (2005). Oral probiotic administration induces interleukin-10 production and prevents spontaneous autoimmune diabetes in the non-obese diabetic mouse. *Diabetologia*.

[67] Mastrangeli G, Corinti S, Butteroni C (2009). Effects of live and inactivated VSL#3 probiotic preparations in the modulation of in vitro and in vivo allergen-induced Th2 responses. *International Archives of Allergy and Immunology*.

[68] Di Giacinto C, Marinaro M, Sanchez M, Strober W, Boirivant M (2005). Probiotics ameliorate recurrent Th1-mediated murine colitis by inducing IL-10 and IL-10-dependent TGF-*β*-bearing regulatory cells. *Journal of Immunology*.

[69] Lavasani S, Dzhambazov B, Nouri M (2010). A novel probiotic mixture exerts a therapeutic effect on experimental autoimmune encephalomyelitis mediated by IL-10 producing regulatory T cells. *PLoS ONE*.

[70] de Moreno de LeBlanc A, Perdigón G (2010). The application of probiotic fermented milks in cancer and intestinal inflammation. *Proceedings of the Nutrition Society*.

[71] Jung BG, Cho SJ, Koh HB, Han DU, Lee BJ (2010). Fermented Maesil (Prunus mume) with probiotics inhibits development of atopic dermatitis-like skin lesions in NC/Nga mice. *Veterinary Dermatology*.

[72] D'Incà R, Barollo M, Scarpa M Rectal administration of Lactobacillus casei DG modifies flora composition and Toll-Like receptor expression in colonic mucosa of patients with mild ulcerative colitis.

[73] Villena J, Barbieri N, Salva S, Herrera M, Alvarez S (2009). Enhanced immune response to pneumococcal infection in malnourished mice nasally treated with heat-killed Lactobacillus casei. *Microbiology and Immunology*.

[74] Li N, Russell WM, Douglas-Escobar M, Hauser N, Lopez M, Neu J (2009). Live and heat-killed lactobacillus rhamnosus GG: effects on proinflammatory and anti-inflammatory cytokines/chemokines in gastrostomy-fed infant rats. *Pediatric Research*.

[75] Foligne B, Nutten S, Grangette C (2007). Correlation between in vitro and in vivo immunomodulatory properties of lactic acid bacteria. *World Journal of Gastroenterology*.

[76] dos Santos LM, Santos MM, de Souza Silva HP, Arantes RME, Nicoli JR, Vieira LQ (2011). Monoassociation with probiotic Lactobacillus delbrueckii UFV-H2b20 stimulates the immune system and protects germfree mice against Listeria monocytogenes infection. *Medical Microbiology and Immunology*.

[77] Sierra S, Lara-Villoslada F, Sempere L, Olivares M, Boza J, Xaus J (2010). Intestinal and immunological effects of daily oral administration of Lactobacillus salivarius CECT5713 to healthy adults. *Anaerobe*.

[78] Generoso SV, Viana M, Santos R (2010). Saccharomyces cerevisiae strain UFMG 905 protects against bacterial translocation, preserves gut barrier integrity and stimulates the immune system in a murine intestinal obstruction model. *Archives of Microbiology*.

[79] Grangette C, Nutten S, Palumbo E (2005). Enhanced antiinflammatory capacity of a Lactobacillus plantarum mutant synthesizing modified teichoic acids. *Proceedings of the National Academy of Sciences of the United States of America*.

[80] Marinho FAV, Pacífico LGG, Miyoshi A (2010). An intranasal administration of Lactococcus lactis strains expressing recombinant interleukin-10 modulates acute allergic airway inflammation in a murine model. *Clinical and Experimental Allergy*.

[81] Steidler L, Hans W, Schotte L (2000). Treatment of murine colitis by Lactococcus lactis secreting interleukin-10. *Science*.

[82] Roselli M, Finamore A, Nuccitelli S (2009). Prevention of TNBS-induced colitis by different Lactobacillus and Bifidobacterium strains is associated with an expansion of *γδ*T and regulatory T cells of intestinal intraepithelial lymphocytes. *Inflammatory Bowel Diseases*.

[83] Mengheri E (2008). Health, probiotics, and inflammation. *Journal of clinical gastroenterology*.

[84] Sybesma W, Hugenholtz J, de Vos WM, Smid EJ (2006). Safe use of genetically modified lactic acid bacteria in food. Bridging the gap between consumers, green groups, and industry. *Electronic Journal of Biotechnology*.

[85] Schotte L, Steidler L, Vandekerckhove J, Remaut E (2000). Secretion of biologically active murine interleukin-10 by Lactococcus lactis. *Enzyme and Microbial Technology*.

[86] Tournoy KG, Kips JC, Pauwels RA (2000). Endogenous interleukin-10 suppresses allergen-induced airway inflammation and nonspecific airway responsiveness. *Clinical and Experimental Allergy*.

[87] Frossard CP, Steidler L, Eigenmann PA (2007). Oral administration of an IL-10-secreting Lactococcus lactis strain prevents food-induced IgE sensitization. *Journal of Allergy and Clinical Immunology*.

[88] Waeytens A, Ferdinande L, Neirynck S (2008). Paracellular entry of interleukin-10 producing Lactococcus lactis in inflamed intestinal mucosa in mice. *Inflammatory Bowel Diseases*.

[89] Loos M, Remaut E, Rottiers P, de Creus A (2009). Genetically engineered Lactococcus lactis secreting murine IL-10 modulates the functions of bone marrow-derived dendritic cells in the presence of LPS. *Scandinavian Journal of Immunology*.

[90] Steidler L, Neirynck S, Huyghebaert N (2003). Biological containment of genetically modified Lactococcus lactis for intestinal delivery of human interleukin 10. *Nature Biotechnology*.

[91] Braat H, Rottiers P, Hommes DW (2006). A phase I trial with transgenic bacteria expressing interleukin-10 in Crohn's disease. *Clinical Gastroenterology and Hepatology*.

[92] Termont S, Vandenbroucke K, Iserentant D (2006). Intracellular accumulation of trehalose protects Lactococcus lactis from freeze-drying damage and bile toxicity and increases gastric acid resistance. *Applied and Environmental Microbiology*.

[93] Huyghebaert N, Vermeire AN, Neirynck S, Steidler L, Remaut E, Remon JP (2005). Development of an enteric-coated formulation containing freeze-dried, viable recombinant Lactococcus lactis for the ileal mucosal delivery of human interleukin-10. *European Journal of Pharmaceutics and Biopharmaceutics*.

[94] Huyghebaert N, Vermeire AN, Neirynck S, Steidler L, Remaut E, Remon JP (2005). Evaluation of extrusion/spheronisation, layering and compaction for the preparation of an oral, multi-particulate formulation of viable, hIL-10 producing Lactococcus lactis. *European Journal of Pharmaceutics and Biopharmaceutics*.

[95] del Carmen S, de Moreno de LeBlanc A, Miyoshi A, Santos Rochat C, Azevedo V, LeBlanc JG (2011). Application of probiotics in the prevention and treatment of ulcerative colitis and other inflammatory bowel diseases. *Ulcers*.

[96] LeBlanc JG, del Carmen S, Miyoshi A (2011). Use of superoxide dismutase and catalase expressing lactic acid bacteria to attenuate TNBS induced Crohn’s disease in mice. *Journal of Biotechnology*.

[97] LeBlanc JG, van Sinderen D, Hugenholtz J, Piard J-C, Sesma F, Savoy de Giori G (2010). Risk assessment of genetically modified lactic acid bacteria using the concept of substantial equivalence. *Current Microbiology*.

